# Caught in the Middle: Combined Impacts of Shark Removal and Coral Loss on the Fish Communities of Coral Reefs

**DOI:** 10.1371/journal.pone.0074648

**Published:** 2013-09-18

**Authors:** Jonathan L. W. Ruppert, Michael J. Travers, Luke L. Smith, Marie-Josée Fortin, Mark G. Meekan

**Affiliations:** 1 Department of Ecology and Evolutionary Biology, University of Toronto, Toronto, Canada; 2 Australian Institute of Marine Science, University of Western Australia Oceans Institute, Crawley, Australia; 3 Woodside Energy, Perth, Australia; Leibniz Center for Tropical Marine Ecology, Germany

## Abstract

Due to human activities, marine and terrestrial ecosystems face a future where disturbances are predicted to occur at a frequency and severity unprecedented in the recent past. Of particular concern is the ability of systems to recover where multiple stressors act simultaneously. We examine this issue in the context of a coral reef ecosystem where increases in stressors, such as fisheries, benthic degradation, cyclones and coral bleaching, are occurring at global scales. By utilizing long-term (decadal) monitoring programs, we examined the combined effects of chronic (removal of sharks) and pulse (cyclones, bleaching) disturbances on the trophic structure of coral reef fishes at two isolated atoll systems off the coast of northwest Australia. We provide evidence consistent with the hypothesis that the loss of sharks can have an impact that propagates down the food chain, potentially contributing to mesopredator release and altering the numbers of primary consumers. Simultaneously, we show how the effects of bottom-up processes of bleaching and cyclones appear to propagate up the food chain through herbivores, planktivores and corallivores, but do not affect carnivores. Because their presence may promote the abundance of herbivores, the removal of sharks by fishing has implications for both natural and anthropogenic disturbances involving the loss of corals, as herbivores are critical to the progress and outcome of coral recovery.

## Introduction

Marine and terrestrial ecosystems are assailed by disturbances that operate as regulators of system structure and function [1]–[4]. For the most part, these are natural perturbations (for example storms or forest fires) that create predictable cycles of destruction and recovery in ecosystem state [2], [5], [6]. However, the effects of human societies on the planet are now so pervasive and far-reaching that through activities such as the burning of fossil fuels, it is predicted that we will ultimately increase the frequency and severity of these disturbances [7], [8]. At the same time, we have introduced new types of anthropogenic disturbances into many ecosystems. The combined effects of these stressors may ultimately affect both ecosystem function and resilience [9], [10].

Coral reef ecosystems offer an ideal model to explore this issue. Reefs are dynamic environments, where pulse disturbances such as coral bleaching, cyclones, crown-of-thorns (*Acanthaster planci*) outbreaks and coral disease are commonplace [3], [11]–[14]. Typically, these alter reef communities in a “bottom-up” manner by causing the death of live corals, which are then overgrown by algae in most places. In turn, this causes changes to the composition and rugosity of the benthic environment, which impacts communities of reef fishes through effects on food resources, settlement and habitat [15]. Where these disturbances are severe but infrequent, corals recover through recruitment and regrowth, a process that can take from one to several decades to complete [11], [16].

Today, many reefs are also afflicted with disturbances that are anthropogenic in origin and chronic in nature. One of the most pervasive of these is the removal of top-order predators such as sharks, a process that has been accelerating throughout the tropics in recent decades [17]–[20]. As sharks have conservative life-history traits (slow growth rates, late sexual maturity, low reproductive output and long gestation), fishing pressure can have a dramatic impact by easily exceeding maximum sustainable yields and the recovery of populations from over-exploitation requires many years [21], [22]. The impact of the loss of sharks on coral reefs is not clear [23]. Ecosystem models give some insight, but provide contrasting evidence of whether sharks play a role in structuring fish communities that is important [24], [25] or relatively minor [26]. Empirical work that has investigated the role of sharks in reef ecosystems has taken a “snapshot” approach when assessing the impact on the fish community, where trophic structure has been compared on reefs with and without sharks at a single instant in time [27]–[29]. This ignores the fact that reef communities respond to a range of disturbances that are natural in origin and operate at a variety of spatial and temporal scales. Such events usually occur at scales greater than entire reefs (10 s–100 s km) and re-structure reef communities in a “bottom-up” manner, in contrast to the “top-down” influence of reef predators [30]. Because in many cases reefs require over a decade to recover from disturbance events [11], any influence of top-down processes in structuring fish communities acts against a background of recovery from these bottom-up agents of change. Thus, if we are to understand the individual and combined effects of both natural and anthropogenic disturbances on reef ecosystems, we require studies with sufficient temporal and spatial scope to disentangle the effects of the loss of sharks as predators and natural disturbances on fish communities.

A second problem in examining the importance of sharks in reef ecosystems concerns the need for accurate and precise estimates of shark abundance. On non-fished reefs, sharks can be abundant [21], [27], [28], even in shallow water (<20 m depth). However, traditional survey techniques, such as underwater visual censuses used to count sharks [31]–[33] are restricted to depths accessible to divers (from the surface to around 30 m depth), which is only a fraction of the range occupied by reef-associated sharks [34]. Furthermore, the behaviour of both the diver and the shark are likely to have an effect on numbers recorded by underwater visual counts [31]. There are well-documented biases in belt transect counts of large-bodied and faster-swimming fish by divers [31]–[33]. In some situations territorial reef sharks may be attracted by the presence of divers on the reef, particularly in locations where the entry of divers into reef waters is a relatively novel event [31]. Given that localities where large numbers of sharks remain are often characterised by their isolation and lack of accessibility to humans, this may be a problem for abundance estimates. Conversely, other places visited by many divers may be avoided by sharks [31], [33]. In either situation, the assumption that reef sharks are indifferent to the presence of divers may bias outcomes of visual censuses.

Here, we examine the relative and combined effects of the loss of sharks as top-order predators due to fishing (a chronic disturbance) and the bottom-up, pulse disturbances of cyclones and bleaching as processes structuring reef fish communities on remote atolls in the eastern Indian Ocean. Because long-term monitoring of fish and coral communities has been conducted on these reefs for over a decade, they provide an ideal ecosystem-scale (hundreds of kilometres), natural experiment to investigate this subject. Our objectives were to examine (1) how fishing changed shark communities in coral reef ecosystems, (2) if such changes impacted the trophic structure of other fish communities, (3) the role of benthic disturbances in structuring fish communities and (4) whether there were any combined impacts of fishing and benthic disturbances on the community structure of fishes.

## Methods

### Study Area

**Figure 1:**
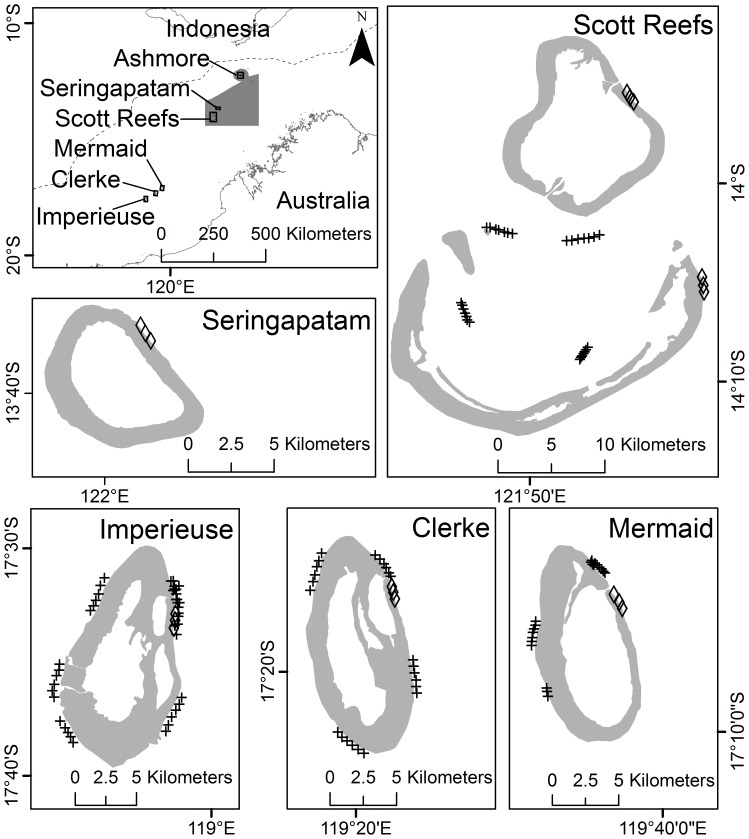
The protected Rowley Shoals (Imperieuse, Clerke and Mermaid Reefs) and fished Scott Reefs (Seringapatam, North Scott and South Scott Reefs). Locations of baited remote underwater video stations (crosses) and long term monitoring program sites (diamonds) are shown on each reef (light grey). The dotted line shows the Australian Economic Exclusive Zone boundary and dark grey denotes the MoU Box 74.

A unique combination of circumstances allowed our study to examine effects of shark removal and benthic disturbances on the trophic structure of coral reef fishes. Since 1994, changes in the abundance and diversity of benthic habitats and fishes have been quantified on the outer reef slopes of two groups of uninhabited, atoll-like coral reefs that lie off the coast of north-western Australia. As this has only involved passive long-term monitoring, no ethical considerations applied in this case. The first of these, the Rowley Shoals (Mermaid, Clerke and Imperieuse Reefs; Figure 1) are marine protected areas (i.e. all forms of fishing are restricted or prohibited), while the second, Scott Reefs (Seringapatam, North and South Scott Reefs; Figure 1) lie within the Australian-Indonesian Memorandum of Understanding Box 74 (MoU74), an area of approximately 50,000 km^2^, where Indonesian fishermen are granted access to the Australian exclusive economic zone to pursue fishing for sharks using traditional techniques [35].

Indonesian fishermen provide a chronic disturbance on the reefs by targeting “banquet” species of high economic value, principally shark (for the shark fin trade), trepang (*Holothuroidea spp.*; sea cucumbers**)** and trochus (*Trochidae spp.*; top snails) in a fishery that has historical origins dating to well before European settlement of Australia [35], [36] (see Text S1 for more details). Australian Customs and border patrol flights (2000–2007) confirm the presence of both legal and illegal Indonesian fishermen in the vicinity of the MoU74 Box, but not as far south as the Rowley Shoals [35]. Biological and physical differences between Scott Reefs and the Rowley Shoals are summarized in Table S1. There were some minor differences in chlorophyll-a and water temperatures (on average around 1°C) between the reefs but there was no evidence that this has led to greater productivity of coral or fish communities at either reef (Table S1). The reef systems were also similar in size. However, there was a greater species richness of fishes at Scott Reefs than the Rowley Shoals, which can be accounted for by the position of the Scott Reefs closer to Indonesia and the centre of reef fish diversity in the Coral Triangle. This difference in diversity was restricted to mostly rare species (i.e. present in <5% of sites) that make only a very minor contribution to patterns of abundance (Table S1).

The Rowley Shoals and Scott Reefs are atoll-like reefs without any significant emergent land that lie over 300 km from the nearest coast. Distances between these groups of reefs and the coast limits any likelihood of larval exchange and genetic evidence suggests that fish communities on the reefs can be largely dependent on self-recruitment [37]. Additionally, tracking studies of grey reef sharks (*Carcharhinus amblyrhynchos*) at the Rowley Shoals have shown that there is little to no movement among reefs within the Shoals [38]. Thus, it is a reasonable assumption that atoll systems are independent of each other in terms of reef-associated fish and shark stocks.

### Benthic Disturbances

**Figure 2:**
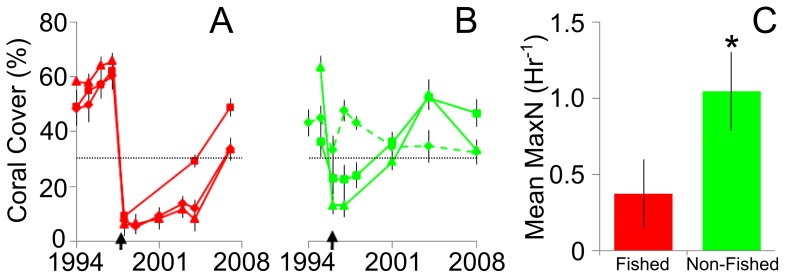
Coral cover and shark density at fished (red) and non-fished (green) reefs. Coral cover is shown for the (A) Scott Reefs and (B) Rowley Shoals, including reefs with (solid line) and without (dashed line) disturbance events. Arrows denote timing of disturbance and the dashed black line denotes the 30% coral cover threshold. (C) Shark density at fished and non-fished reefs measured as the maximum number of each species seen together at any one time (MaxN) per hour of tape. Values are the means ±95% confidence intervals. *p<0.05 for permuted *t*-test.

Both the Scott Reefs and Rowley Shoals experienced catastrophic pulse disturbances in the late 1990s. At the Scott Reefs, bleaching reduced coral cover from *c.* 60% to <10%, while similar reductions in coral cover occurred at two of three reefs of the Rowley Shoals after a Category 5 cyclone (Figure 2A and 2B). Corals killed directly or indirectly by these pulse disturbances were overgrown by turfing algae, but coral cover returned to near pre-disturbance levels in the following decade. We used a threshold of <30% coral cover to classify reefs as pulse disturbed (impacted and/or recovering) or ≥30% coral cover as non-disturbed (not impacted or recovered; see Figure 2A and 2B). This threshold was chosen because coral cover averaged around 30% for most reefs during the monitoring period (Table S1) and this level of cover has been used to define “healthy” reefs worldwide [1], [39].

### Survey Data

Sharks were sampled using BRUVS [40] at Mermaid (*n* = 28) and Scott Reefs (North and South Scott only; *n* = 28) during June 2003 and at Clerke (*n* = 24) and Imperieuse (*n* = 42) Reefs in the Rowley Shoals in October 2004 (Figure 1). Generally, sampling occurred at 3–4 sites on the outer reef slope of each reef. At each site, six BRUVS were deployed on the reef slope during the day for approximately one hour of soak time, with each replicate being separated by at least 500 m (10–60 m depth range; Figure 1). All deployments were spread throughout daylight hours from 07∶00–16∶00 hrs. Interrogation of each tape provided the maximum number of each species seen together in any one time on the whole tape (MaxN). Here, we report standardized shark abundances as MaxN per hour.

Data on fish abundance and benthic cover were collected by the Australian Institute of Marine Science Long Term Monitoring Program (LTMP) usually in October of 1994–2008 [41], [42]. Censuses of non-cryptic, adult (1+ year old) fishes were conducted at 3 sites on each reef along 5 fixed transects that were each 50 m in length and deployed along the 6–9 m depth contour of the reef slope (Figure 1). The start of each transect was separated from the end of the preceding transect by 10 m. Larger and more mobile species (e.g. Lutjanidae, Lethrinidae) were counted within 2.5 m either side of the transect tape and site-attached species (e.g. Pomacentridae) were counted on a return swim within 0.5 m either side of the belt transect. Fishes were identified to species and classified into five trophic groups: carnivores, herbivores, planktivores, corallivores and detritivores (Table S2). Prior to analysis of the resulting data sets, we removed rare species (defined as present in <5% of sites) to reduce the influence of zeroes in multivariate analysis [43]. A total of 112 species remained of which the most diverse were carnivores (26 species including representatives of the families Chaetodontidae, Epinephelidae, Labridae, Lutjanidae, Lethrinidae and Zanclidae) and herbivores (43 species including representatives of the families Acanthuridae, Pomacentridae, Siganidae and subfamily Scarinae), while corallivores (20 species, representatives of the Chaetodontidae and Pomacentridae), detritivores (5 species, all from the Acanthuridae) and planktivores (18 species including representatives of the Lutjanidae, Pomacentridae and Acanthuridae) tended to be dominated by only one family (Table S2). The benthos was sampled along the same transects using a video camera that recorded a 0.5 m wide path along the transect line. Percentage cover estimates of 19 different categories of benthos were extracted using a video frame analysis [42]. These categories were selected based on relative contribution to total cover and their importance to habitat and dietary requirements of reef fishes.

### Analysis

To determine the extent to which differences in habitats between reef systems contributed to patterns in fish communities, we used a forward selection procedure to select 15 of 19 habitat categories (encrusting coral, *Isopora*, soft coral, corymbose *Acropora*, digitate *Acropora*, Pocilloporidae, sponge, massive coral, foliose, macro algae, turf-coralline algae, other algae, sand/rubble, other coral, and other) in order to create a habitat model (see [44] and see Text S1 for more details). A fishing model related to the presence or absence of the Indonesian fishery was then constructed, along with a model that included both habitat and fishing. We then used a sequence of Redundancy Analysis (RDA), permutation tests (*n* = 999) and variance partitioning to estimate the amount of variation in community structure that could be accounted for by differences in habitat, fishing and an interaction between these factors [44], [45]. Total variation of the fish community was decomposed into habitat, fishing, shared components and unexplained variation. The amount of variation in the fish community that was uniquely attributable to habitat and fishing components was then identified using an adjusted *R* ^2^ [45]. This procedure used the *anova*, *packfor*, *varpart* and *rda* libraries in the *vegan* package of R Statistical Computing [46].

Principal Components Analysis (PCA) and RDA were used to investigate differences in benthic and fish assemblages among four treatment groups: fished/disturbed (*n = *30), non-fished/disturbed (*n* = 21), fished/non-disturbed (*n* = 46) and non-fished/non-disturbed (*n* = 29). In this case, fishing represented a chronic disturbance at the Scott Reefs that has been occurring for centuries, while the reef fishes at the Rowley Shoals are protected from fishing (Figure 1). A disturbance treatment included fish and benthic communities where coral was reduced below 30% cover after a cyclone that occurred on non-fished reefs in 1996 and bleaching that occurred on fished reefs in 1998 (Figure 2A and 2B). Species composition was described by the abundance of the five trophic groups (carnivores, herbivores, detritivores, corallivores and planktivores) in the RDA. We pooled pre- and post-disturbance fish communities together as we found that on both fished and non-fished reefs, the densities of trophic groups after the recovery of coral was similar to that occurring prior to pulse disturbances over the period of 1994–2008 [47]. This procedure used the *cca* and *anova* libraries in the *vegan* package of R Statistical Computing [46].

Comparisons of shark abundance between fished (Scott Reefs) and non-fished (Rowley Shoals) reefs were conducted using R Statistical Computing [46] with a one-tailed permuted (*n* = 9999) *t*-test that accommodated non-parametric data with unequal sample sizes [48]. Shark abundances were surveyed only during the years 2003 and 2004. However, due to their conservative life history traits (longevity, late age of maturation, low fertility) a snapshot for this group was thought to be more appropriate than for reef fishes, which have much faster turn-over times of populations. Furthermore, very low abundances of reef sharks at the Scott Reefs were noted in surveys in 1998 near the start of our study [49] and again in 2010–11 [50]. Thus, we suggest that the abundance estimates of sharks shown here were representative of the period of 1994–2008. The Scott Reefs had fewer BRUVS samples, so we compared shark abundance at Scott Reefs with data available from BRUVS surveys of Ashmore Reef (*n* = 46), another fished reef within the MoU74 box using a two-tailed permuted *t*-test (*t*_72_ = 0.76, *p* = 1.0). As there was no significant difference in shark abundance between these reefs, we used data from Scott Reefs for subsequent analyses.

Permuted ANOVAs were conducted using *adonis* function in the *vegan* package of R Statistical Computing [46]. To test for the fixed-effects of fishing, disturbance and their interaction we used permuted ANOVAs (*n* = 9999). Further pairwise comparisons using two-tailed permuted *t*-tests (*n* = 9999) were conducted to test for fishing and disturbance effects between the four treatment groups. As Euclidean distances were used in permuted ANOVAs, abundances were Hellinger transformed prior to testing [51]. Bonferroni corrections were used to adjust significance levels for multiple tests [52].

## Results

A majority of the differences in benthic composition at both fished and non-fished sites were related to coral and algae cover (44.7% of variation), which was associated with the pulse disturbance events observed on all reefs (Figure S1). However, the PCA demonstrated that there were differences related to the severity of disturbances, where fished reefs had more algae and less coral following bleaching than non-fished reefs after the cyclone event (Figure S1). Further, some other differences in the benthic community were apparent between reef systems, which could be attributed to a few benthic groups (mainly macro algae, sponges, *Isopora*, corymbose *Acropora*). These groups made only a small contribution to patterns in benthic composition (only 15.8% of variation; Figure S1) and collectively represented less than 13% of average benthic cover across sites.

Variance partitioning and permutation tests revealed that habitat (i.e. benthic cover) significantly (*p*<0.001) and uniquely explained 23.4% of variation in the fish community across all sites. Further, the presence or absence of fishing significantly (*p*<0.001) and uniquely explained 13.8% of variation in the fish community across all sites. Finally, the model that combined habitat and fishing was significant (*p*<0.001) and was able to explain 60.3% of variation in the fish community (23.4% of the variance was explained by the habitat, 13.8% was explained by fishing, and 23.1% of variation was shared between habitat and fishing effects). Thus, both habitat and fishing uniquely and interactively contributed to patterns in reef fish communities across the sites.

As fishing was significantly associated with patterns in reef fish communities we examined these patterns in more detail by first investigating differences in shark density. The BRUVS sampling showed that abundances of reef sharks (notably silvertip, *Carcharhinus albimarginatus* and grey reef, *C. amblyrhynchos*) at the protected Rowley Shoals were approximately three times those occurring on the fished Scott Reefs (*t*_96_ = 3.86, *p* = 0.0175; Figure 2C).

**Figure 3:**
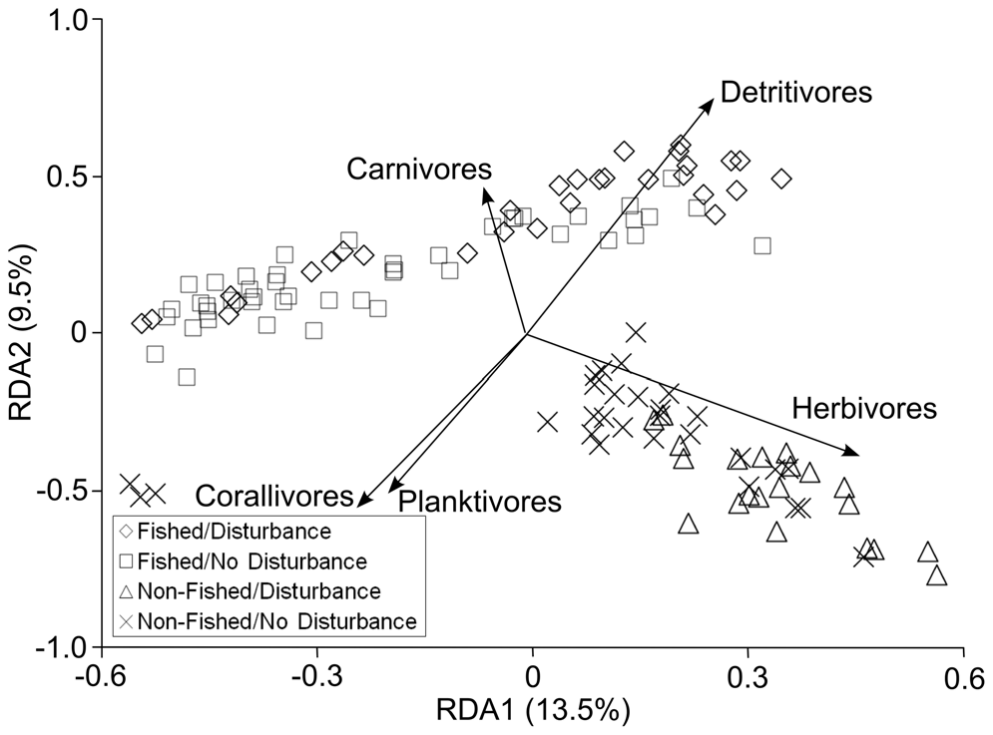
Redundancy analysis of species composition (n = 112) for five trophic groups (carnivore, herbivore, detritivore, corallivore and planktivore) of reef fishes. Sites have been classified by the four treatments and the variation explained by each axis is shown.

**Figure 4:**
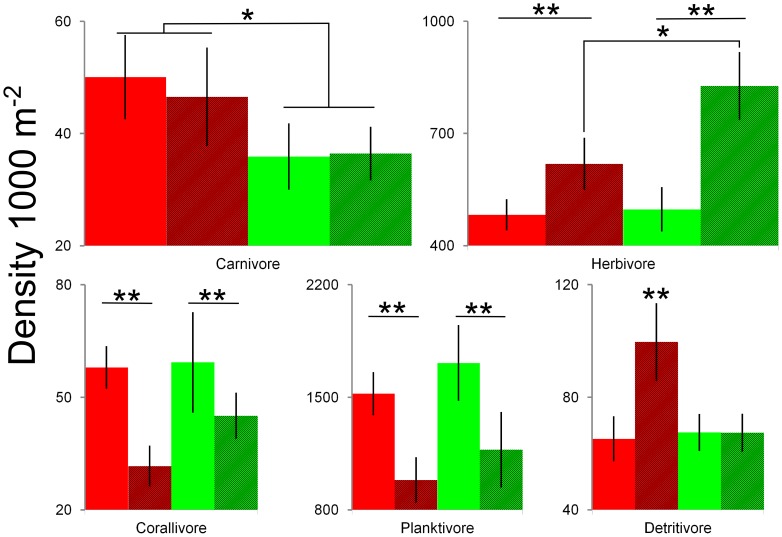
Mean density of trophic groups (±95% confidence intervals) for fished (red) and non-fished (green) reefs. The density of trophic groups across fished, non-fished, disturbed (stippled bars) and non-disturbed (solid bars) reefs are shown. **p<0.01 and *p<0.05 for permuted ANOVAs and permuted *t*-tests.

Associated with changes in shark densities were clear differences in assemblage and trophic structure between fished and non-fished reef systems, notably in the abundances of carnivores and herbivores (Figure 3). Assemblages on the fished Scott Reefs had significantly greater numbers of mid-sized carnivores than the protected Rowley Shoals (Figure 4 and Table S3). These differences were largely attributed to changes in numbers of lutjanids along with lethrinids, epinephelids and some chaetodontids. Multiple species from these families contributed to this pattern (Figure S2). Densities of primary consumers also differed between reefs, so that herbivorous fishes were significantly more abundant at the protected Rowley Shoals than at the Scott Reefs following a pulse disturbance event (Figure 4 and Table S3). Again, these differences were attributable to representatives of most of the major families of herbivores, including scarine labrids, acanthurids and pomacentrids (Figure S3). We observed no significant differences in the densities of corallivorous and planktivorous fishes between fished and non-fished reefs (Figure 4 and Table S3).

Variations in the densities of planktivores and corallivores were related to habitat rather than fishing effects (Figure 3). Densities of these trophic groups were significantly and negatively correlated with the timing of pulse disturbances (bleaching, cyclones; Figure 4 and Table S3) so that after the loss of coral cover, densities declined. In contrast, densities of herbivores increased significantly after coral cover declined (Figure 4). Herbivores were also the only group to demonstrate significant differences in density between fished and non-fished reefs in response to a pulse disturbance event. The effects of bottom-up, pulse disturbances did not appear to propagate further up the food chain, as abundances of carnivores remained constant throughout the changes in coral cover (Figure 4).

Finally, we found that 23.1% of variation in the fish community was shared between habitat and fishing effects. In part, this may be due to a synergistic effect of shark removal and coral loss on detritivores (Figure 4 and Table S3), where this trophic group increased in abundance during a pulse disturbance to a far greater degree on fished rather than non-fished reefs.

## Discussion

Top-order predators such as sharks have the capacity to directly alter the composition of fish communities through consumption of prey or by inducing costly behavioural-risk effects [53]. For these reasons, it might be expected that sharks should have a strong influence on the species composition, biomass and trophic structure of prey assemblages [28], [29]. Nevertheless, demonstrating such effects has been problematic, usually because the loss of sharks is accompanied by a wide range of other anthropogenic disturbances. These include the exploitation of fishes and invertebrates at lower trophic levels, a process coined “fishing down the food chain” [54] and ecosystem degradation through pollution, eutrophication and habitat loss, particularly where atolls are inhabited by growing human populations [29], [55]. Furthermore, many previous studies are limited in temporal scope and compare fish and benthic community structure across a gradient of shark abundance on reefs at only a single instant in time [27]–[29]. This ignores the fact that coral communities are dynamic and that even pristine reefs are invariably in a state of flux between impact and recovery from natural, pulse disturbances that alter the structure of fish assemblages in a bottom-up manner. As our study atolls are uninhabited and the targets of fishing by Indonesians are largely limited to “banquet” species of high economic value, such as sharks [35], we were able to investigate the potential effects of loss of sharks on community structure, without the confounding effects of other anthropogenic disturbances. Moreover, as we monitored changes in fish and benthic assemblages for more than a decade, we were able to extract the effects of shark fishing from the background of changes in benthic community structure related to cyclones and bleaching.

Although this unique set of circumstances offers, for the first time, an opportunity to examine the effects of chronic and pulse disturbances on a coral reef ecosystem, our results must be considered within the context and limitations of a natural experiment. Because all such studies are correlative in nature, in most cases alternative explanations for patterns cannot be excluded. Bearing this caveat in mind, using variance partitioning and permuted ANOVAs we found evidence that both shark fishing and habitat were significantly and uniquely associated with the composition and trophic structure of reef fish assemblages.

Fishing was significantly associated with declines in shark numbers and was also associated with high abundances of smaller, mesopredators on our study reefs (Figure 4). This observation was consistent with the phenomenon of “mesopredator release” where smaller, secondary carnivores become very abundant as a consequence of the removal of larger, top-order predators from an ecosystem. Numerous examples of this process have been documented in both terrestrial and marine environments [56]–[60]. At the Scott Reefs, mesopredators were generally mid-sized (15–40 cm; mostly representatives of the Lutjanidae and Lethrinidae) species that consumed both fishes and invertebrates (Figure S2). Abundances of mesopredators appeared to be independent of bottom-up changes in coral habitats, as numbers of this guild did not alter during the impact or recovery from the bleaching event at Scott Reef or the cyclone at the Rowley Shoals that removed up to 80% of the cover of live coral in shallow (<30 m depth) water (see Figure 2 and Figure 4). Herbivorous fishes were less abundant at the fished Scott Reefs than at the unfished Rowley Shoals. Potentially, this could indicate that a trophic cascade had occurred, so that the reduction in numbers of sharks as top-order predators may have not only affected the smaller carnivores, but also herbivorous fishes (from multiple genera; Figure 4 and Figure S3). In contrast, we could find no evidence that abundances of corallivores and planktivores differed between fished and unfished reefs. Changes in habitat brought about by the loss of live coral during pulse disturbances appeared to be the principal factor driving variation in the composition and numbers of these groups of consumers. Abundances of both corallivores and planktivores declined with the loss of live coral, which probably reflected a reduction in food resources, habitat and settlement sites for these reef fishes [15], [61], [62]. In contrast, numbers of herbivorous fishes were positively correlated with the increasing algal cover that replaced corals in the aftermath of the cyclone and bleaching events (Figure 4). Such correlations between loss of coral cover and changes in the abundance of these trophic groups are typical of those recorded by many earlier studies [63], [64].

Disturbances can act synergistically, additively or antagonistically on the abundances of animals within an ecosystem [10]. In our study, bottom-up and top-down disturbances may have had a synergistic effect on abundances of detritivores, as the change in density of this group was not simply a sum (additive) or less than additive sum (antagonistic) of individual stressors [10]. Fishes of this trophic group (mostly surgeonfishes of the genus *Ctenochaetus*) were significantly more abundant on the fished Scott Reefs after the impact of bleaching than during undisturbed phases. In contrast, they did not differ in abundance during impact and undisturbed phases on the unfished Rowley Shoals. This result implies that the reduction in shark numbers may directly or indirectly allow these species to take advantage of the increase in detrital material trapped by turfing algae that overgrew dead corals during the disturbance at the Scott Reefs.

We did, however, find that there were a number of differences in benthic habitats between the reef systems. As these were collinear with any effect of fishing (i.e. reefs differed in both fishing and benthic habitats) we cannot completely discount these as alternative explanations for some of the patterns we found in fish communities on reefs with and without reduced numbers of sharks. For the most part, such habitat differences were relatively small. While our PCA identified macro algae, sponges, *Isopora* and corymbose *Acropora* as benthic groups that differed between reef systems (Figure S1), these contributed to 15.8% of variation in the data set and in total only represented less than 13% of total benthic cover. Of potentially greater importance, the PCA did show evidence that the bleaching event at Scott Reefs was more severe in terms of removal of live coral than the cyclone at the Rowley Shoals. This may have benefitted detritivores at the Scott Reefs by creating more resources (Figure S1), resulting in the greater numbers of this trophic group during the disturbance phase on these reefs. Such a hypothesis is difficult to reconcile with our observations, since we would expect that a greater loss of live coral and thus the presence of more algae should also result in greater numbers of herbivores at the Scott Reefs than the Rowley Shoals. In fact, we recorded the opposite pattern, with fewer herbivores at Scott Reefs than the Rowley Shoals.

Some of our results might also reflect other, more fundamental differences in the nature of the disturbances between reef systems. For example, wave action caused by cyclones breaks up coral skeletons, reducing three-dimensional structure of the reef [62]. In contrast, bleaching removes only the outer layer of live coral, leaving the skeleton and the habitat intact. Potentially, this could explain differences in abundance of detritivores between Scott Reefs and the Rowley Shoals, since the bleaching at Scott Reefs may have produced reefs that trapped more detritus, increasing resources for this trophic group. However, such effects are short-lived, lasting no more than a few months. After this time bioeroders and wave action create significant structural collapse of coral skeletons, so that the ultimate effects of both types of disturbance rapidly become very similar [65]. We found that the increased abundance of detritivores at the Scott Reefs was not an ephemeral event, but was sustained over the many years that the reefs required to recover from severe bleaching [47] suggesting that differences in the effects of the initial disturbance event could not account for this result.

Finally, another possibility is that differences in numbers of some trophic groups between fished and unfished reefs may be simply a result of random variation in patterns of larval supply. For surgeonfishes, rare strong pulses in recruitment (greater by orders of magnitude than background levels) can be a feature of their biology on isolated reefs and atolls [66]. Given that these fishes make up the majority of the detritivore group at both our study reefs and we did not monitor recruitment, we cannot exclude the possibility that rather than a synergistic effect of the loss of sharks and a pulse disturbance, the increase in abundance of detritivores at Scott Reef after the bleaching was due to one of these sporadic recruitment events that coincided with the loss of coral cover.

One interpretation of the correlation between low numbers of sharks and herbivorous fishes on our study reefs is that this is evidence of a trophic cascade. These occur when changes in the abundance of higher-order predators directly and/or indirectly affect species at a number of lower trophic levels in a food web. Such cascades are well-recognised in marine systems, with examples involving reductions in the numbers of sharks, lobsters, seastars and sea otters as top-order predators causing fundamental changes in the structure and function of temperate marine ecosystems where they formerly occurred [60], [67], [68]. Despite the correlation between shark abundance and herbivores, we could not show the mechanism that linked these trophic levels. This is perhaps not surprising, given that high species diversity, intraguild predation and wide niche-breaths of diet (e.g. omnivory) are typical traits of assemblages of coral reef fishes. Thus, the precise impacts of predators on reefs can be very difficult to discern [23], [24], [56]. For example, in the Caribbean [59], an increase in reef fish mesopredators resulted in higher predation rates on fish recruits, with this effect not being limited to a single trophic group, but expressed across all abundant species of recruits, ranging from mobile herbivores (*Scarinae*) to damselfishes (*Pomacentridae*).

Irrespective of the mechanism involved, if a link does exist between the abundances of sharks and herbivores, then this has important implications for coral reef ecosystems. Herbivorous fishes are fundamental to the dynamics of communities on reefs, since their feeding reduces algal cover and allows corals more space to colonize and grow in benthic habitats [1], [60]. This role is not limited to any particular type of herbivore (e.g. scraper, roving grazer, territorial grazer); rather all feeding modes are thought to be important [69]. Because bottom-up disturbances that kill live coral result in an increased cover of algae, our results suggest that top-order predators may have a role in determining the rate of recovery of reefs from these events.

Although we may soon lack any practical ability to affect the frequency of bottom-up disturbances to coral reefs where these are driven by climate change, this is not the case with the loss of reef sharks. Tracking studies show that reef sharks can maintain a high degree of site fidelity around coral reefs [38], [70], so that options such as marine protected areas can be an effective means to conserve numbers of these top-order predators [71]. Healthy populations of reef sharks should be a key target of management strategies that seek to ensure the future resilience of coral reef ecosystems.

## Supporting Information

Figure S1 **Principal components analysis of the benthic composition of 19 different classes of coral, algae, sponge, and other benthos among sites.** Benthic cover types contributing the most to patterns are denoted in black, while others are shown in the middle of the plot in grey. Sites have been coded by the four treatments (see key). The amount of variation explained by each axis is shown.
(TIF)

Figure S2 **PCA biplot of fish abundances by genus in the carnivore trophic group.** The sites were coded by each of the four treatments and the 15 genera that made up the carnivore group are shown. The amount of variation explained by each axis is shown.
(TIF)

Figure S3 **PCA biplot of abundances of fish by genus in the herbivore trophic group.** The 12 genera that make up the herbivore group are shown on the figure and the sites were coded by each of the four treatments. The amount of variation explained by each axis is shown.
(TIF)

Table S1 **A summary of anthropogenic, reef metrics, environmental and biotic factors at protected and fished reefs.** Protected sites included Mermaid, Clerke and Imperieuse Reefs. Fished sites included South and North Scott, Seringapatam and Ashmore Reefs.
(DOCX)

Table S2 **Species composition of the five trophic groups (carnivore, herbivore, detritivore, planktivore and corallivore) used in our study.** The list is alphabetical by family and species. Those classified as corallivores included both obligate and facultative coral feeders [11], [18]. Herbivores were classified according to Green and Bellwood (2009) while detritivores (including epilithic algal matrix feeders) followed Wilson et al. (2003). Planktivores and carnivores followed Froese & Pauly (2011) [19]–[21]. Only those species present in more than 5% of sites were included in this list.
(DOCX)

Table S3 **Summary of statistical tests to evaluate fishing, disturbance and interactive effects on densities of trophic groups.** Fishing, disturbance and interaction effects were evaluated using a permuted two-way ANOVA. Permuted *t*-tests were used to conduct contrasts. *p*-values were Bonferroni corrected.
(DOCX)

Text S1 (DOCX)
