# Supplementary figures and images for: Caught in the Middle: Combined Impacts of Shark Removal and Coral Loss on the Fish Communities of Coral Reefs
Source: PLoS One. 2013 Sep 18;8(9):e74648. doi: 10.1371/journal.pone.0074648 (PMC3776739; doi:10.1371/journal.pone.0074648)

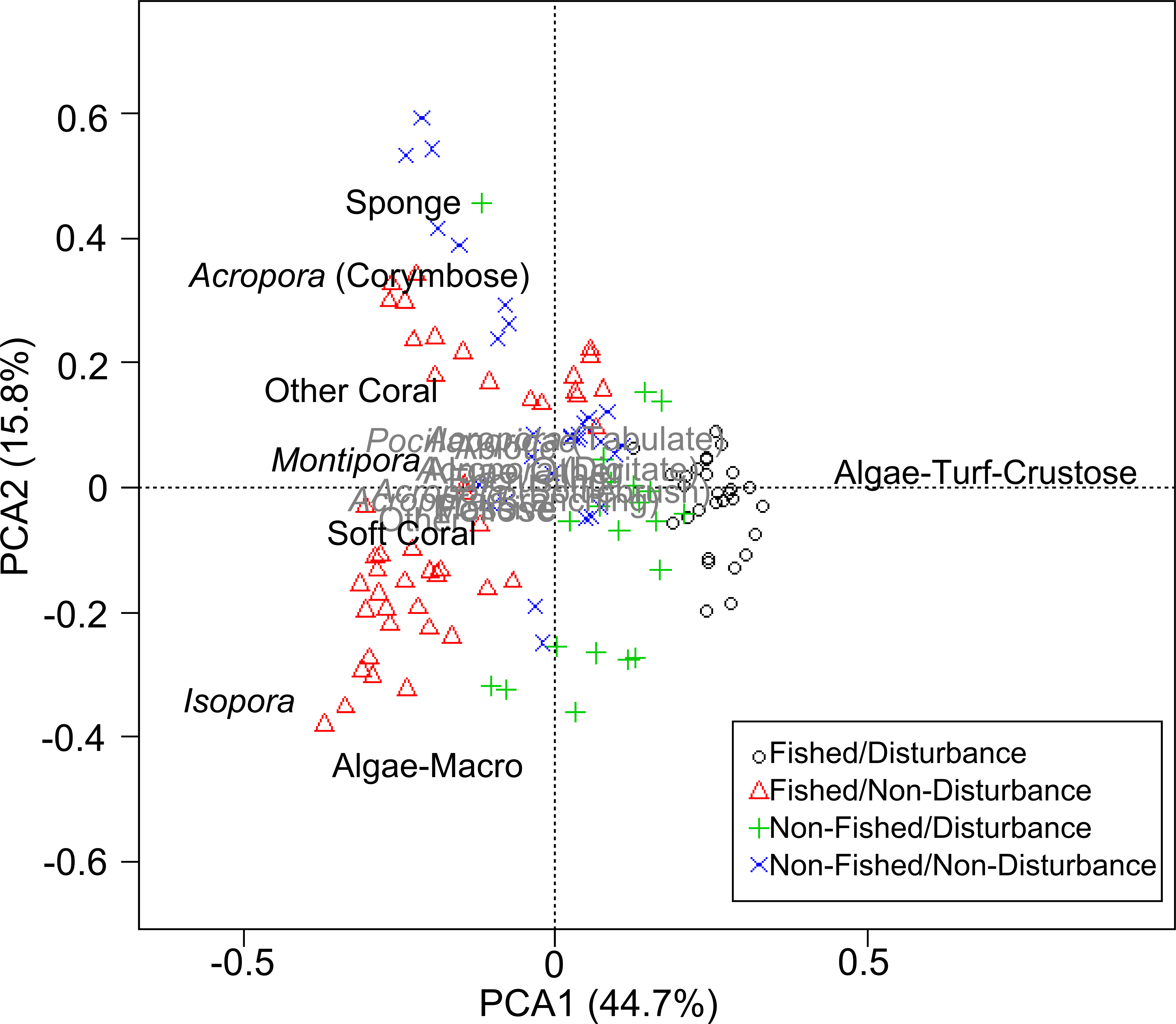

Supplement: Figure S1 — Principal components analysis of the benthic composition of 19 different classes of coral, algae, sponge, and other benthos among sites. Benthic cover types contributing the most to patterns are denoted in black, while others are shown in the middle of the plot in grey. Sites have been coded by the four treatments (see key). The amount of variation explained by each axis is shown. (TIF) [file pone.0074648.s001.tif]

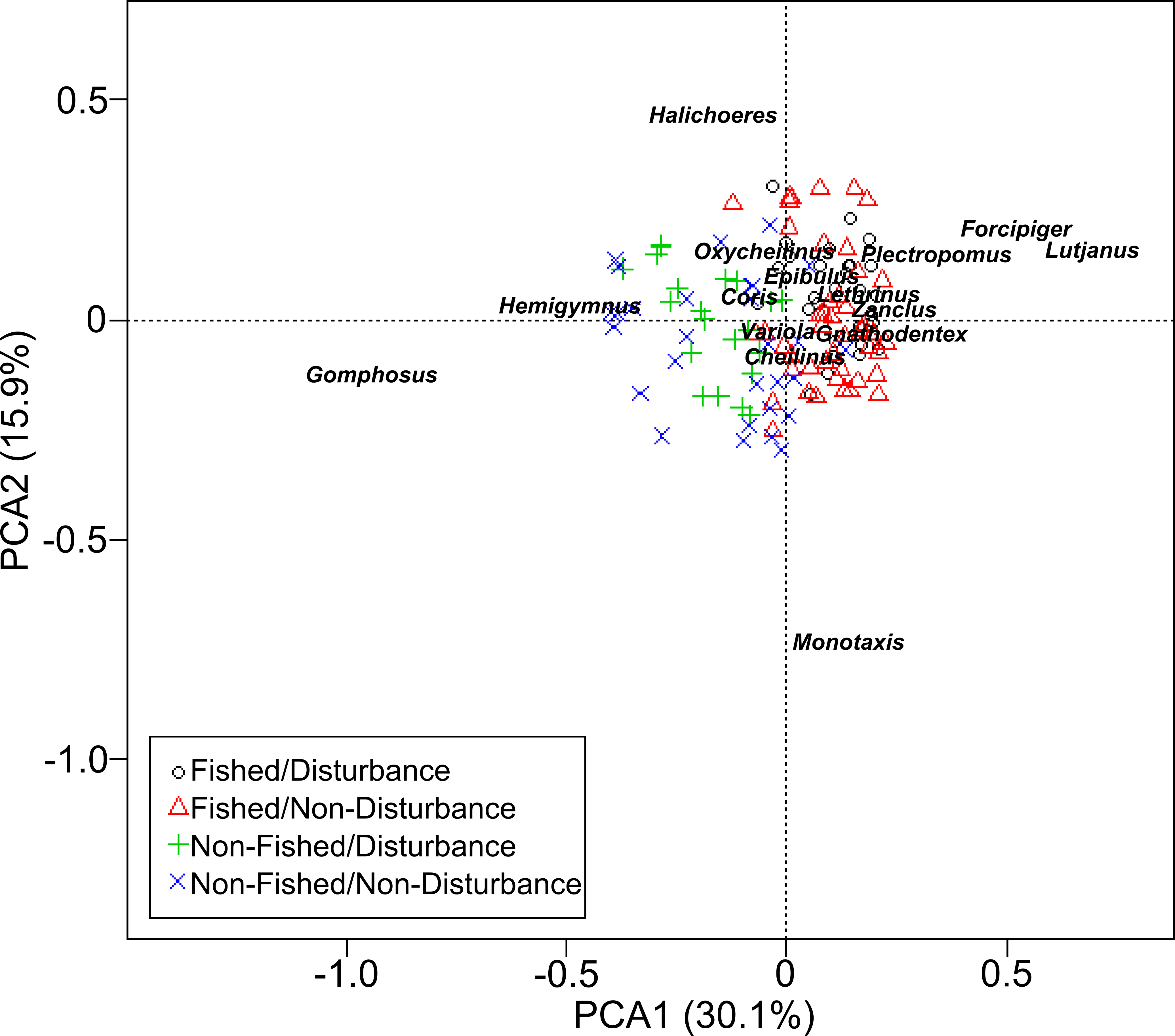

Supplement: Figure S2 — PCA biplot of fish abundances by genus in the carnivore trophic group. The sites were coded by each of the four treatments and the 15 genera that made up the carnivore group are shown. The amount of variation explained by each axis is shown. (TIF) [file pone.0074648.s002.tif]

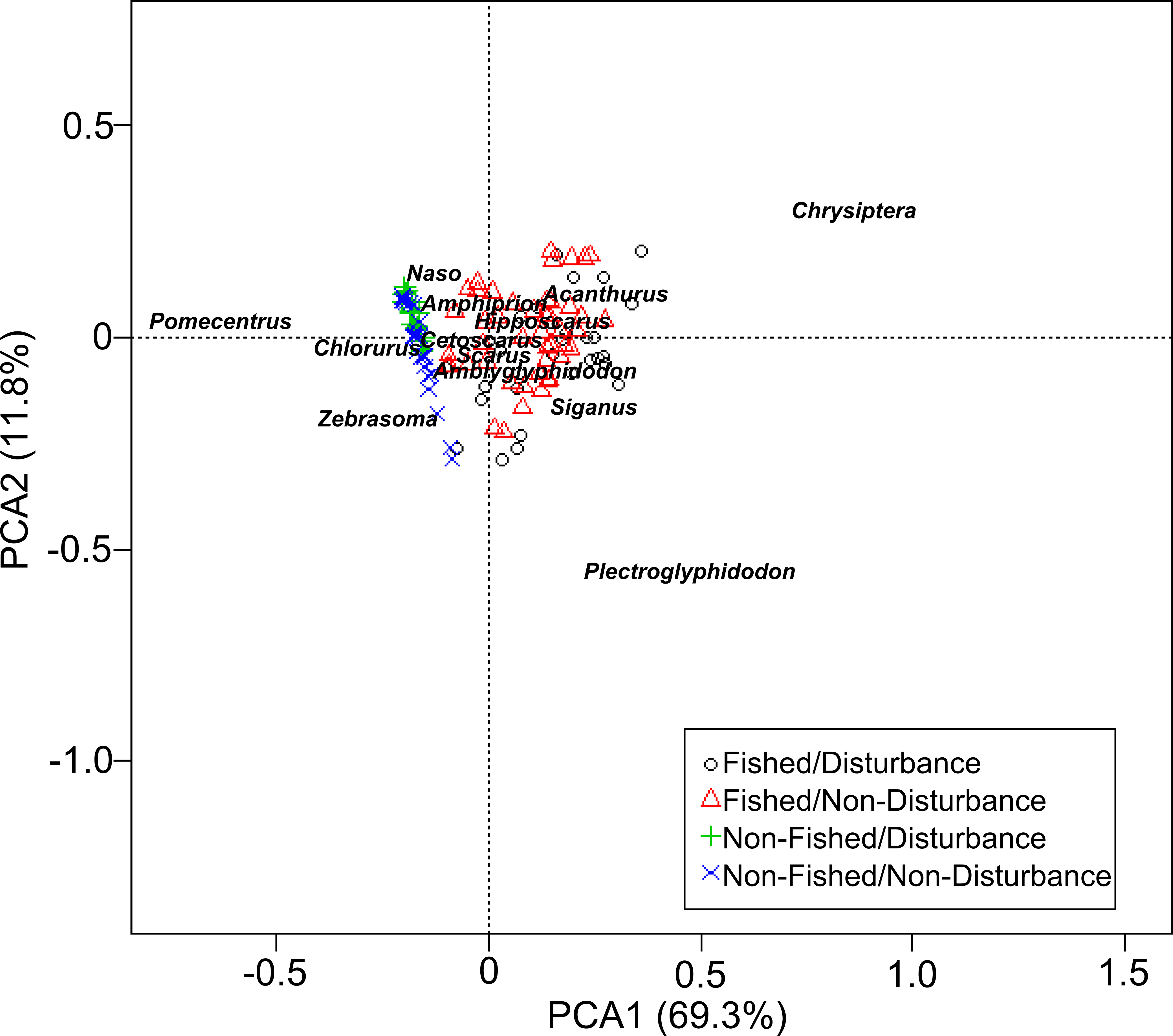

Supplement: Figure S3 — PCA biplot of abundances of fish by genus in the herbivore trophic group. The 12 genera that make up the herbivore group are shown on the figure and the sites were coded by each of the four treatments. The amount of variation explained by each axis is shown. (TIF) [file pone.0074648.s003.tif]
