# Supplementary material for: Caught in the Middle: Combined Impacts of Shark Removal and Coral Loss on the Fish Communities of Coral Reefs
Source: PLoS One. 2013 Sep 18;8(9):e74648. doi: 10.1371/journal.pone.0074648 (PMC3776739; doi:10.1371/journal.pone.0074648)
Supplement: Table S1 — A summary of anthropogenic, reef metrics, environmental and biotic factors at protected and fished reefs. Protected sites included Mermaid, Clerke and Imperieuse Reefs. Fished sites included South and North Scott, Seringapatam and Ashmore Reefs. (DOCX) [file pone.0074648.s004.docx]

**Table S1**.

| **Reef** | **Anthropogenic Factors** | | | |  | **Reef Metrics** | |
| --- | --- | --- | --- | --- | --- | --- | --- |
|  | **Fishing** | **Management** | **Year Established** | **Population** |  | **Area** (km^2^)** | **Perimeter** (km)** |
| Mermaid | Prohibited | Commonwealth Marine Reserve | 1991 | 0 |  | 42.4 | 36.25 |
| Clerke | Prohibited | DEC; Marine Park | 1990 | 0 |  | 58.9 | 38.54 |
| Imperieuse | Prohibited | DEC; Marine Park | 1990 | 0 |  | 72.7 | 43.18 |
| South Scott | Artisanal Fishing | MoU74 Box | 1974 | 0 |  | 99.0 | 108.60 |
| North Scott | Artisanal Fishing | MoU74 Box | 1974 | 0 |  | 56.0 | 48.90 |
| Seringapatam | Artisanal Fishing | MoU74 Box | 1974 | 0 |  | 25.0 | 26.74 |
| Ashmore | Artisanal Fishing | MoU74 Box; Marine Nature Reserve | 1983 | 0 |  | 179.3 | 101.45 |

**Table S1** (Cont’d).

| **Reef** | **Environment Factors** | | | |  | **Biota** | | | | |
| --- | --- | --- | --- | --- | --- | --- | --- | --- | --- | --- |
|  | **Time-Averaged SST (ºC)** | **Time-Averaged Chl a (mg/m^3^)** | **Recent Bleaching Events** | **Recent Cycloninc Events** |  | **Number of Fish Species** | **Rare Species (<5% of sites)** | **Mean Abundance (m^2^)** | **Mean Coral Cover ± SE** | **Mean Algal Cover ± SE** |
| Mermaid | 28.36 | 0.76 |  | 1996 |  | 84 | 0 | 2.84 ± 0.72 | 38.54 ± 6.82 | 32.6 ± 22.56 |
| Clerke | 28.26 | 0.90 |  | 1996 |  | 89 | 0 | 3.31 ± 1.0 | 29.95 ± 10.1 | 35.39 ± 22.78 |
| Imperieuse | 28.09 | 0.20 |  | 1996 |  | 86 | 0 | 3.04 ± 0.94 | 29.8 ± 18.9 | 49.41 ± 23.41 |
| South Scott | 29.04 | 0.28 | 1998 |  |  | 127 | 18 | 2.76 ± 0.73 | 32.81 ± 25.93 | 35.97 ± 26.18 |
| North Scott | 29.10 | 0.61 | 1998 |  |  | 117 | 19 | 2.65 ± 0.73 | 32.25 ± 21.78 | 32.73 ± 21.17 |
| Seringapatam | 29.10 | 0.31 | 1998 |  |  | 134 | 10 | 2.63 ± 0.53 | 45.15 ± 18.38 | 28.5 ± 18.56 |
| Ashmore | - | - | - | - |  | - | - | - | - | - |

* Sea Surface Temperature (SST; 2001 - 2008) and Chlorophyll a (Chl a; 1997 - 2008 ) were derived from remotely sensed imagery produced with the Giovanni online data system, developed and maintained by the NASA GES DISC at 4km resolution.

**Area and perimeter derived from ARCGIS layers
