# Supplementary material for: Caught in the Middle: Combined Impacts of Shark Removal and Coral Loss on the Fish Communities of Coral Reefs
Source: PLoS One. 2013 Sep 18;8(9):e74648. doi: 10.1371/journal.pone.0074648 (PMC3776739; doi:10.1371/journal.pone.0074648)
Supplement: Table S2 — Species composition of the five trophic groups (carnivore, herbivore, detritivore, planktivore and corallivore) used in our study. The list is alphabetical by family and species. Those classified as corallivores included both obligate and facultative coral feeders [11], [18]. Herbivores were classified according to Green and Bellwood (2009) while detritivores (including epilithic algal matrix feeders) followed Wilson et al. (2003). Planktivores and carnivores followed Froese & Pauly (2011) [19]–[21]. Only those species present in more than 5% of sites were included in this list. (DOCX) [file pone.0074648.s005.docx]

**Table S2**.

| **Family** | **Species** | **Trophic Group** |
| --- | --- | --- |
| Acanthuridae | *Acanthurus auranticavus* | Herbivore |
|  | *Acanthurus blochii* | Detritivore |
|  | *Acanthurus dussumieri* | Detritivore |
|  | *Acanthurus fowleri* | Herbivore |
|  | *Acanthurus leucocheilus* | Detritivore |
|  | *Acanthurus lineatus* | Herbivore |
|  | *Acanthurus nigricans* | Herbivore |
|  | *Acanthurus nigricauda* | Herbivore |
|  | *Acanthurus nigrofuscus* | Herbivore |
|  | *Acanthurus olivaceus* | Detritivore |
|  | *Acanthurus pyroferus* | Herbivore |
|  | *Acanthurus thompsoni* | Planktivore |
|  | *Ctenochaetus spp* | Detritivore |
|  | *Naso brevirostris* | Planktivore |
|  | *Naso hexacanthus* | Planktivore |
|  | *Naso lituratus* | Herbivore |
|  | *Naso unicornis* | Herbivore |
|  | *Naso vlamingii* | Planktivore |
|  | *Zebrasoma scopas* | Herbivore |
|  | *Zebrasoma veliferum* | Herbivore |
| Chaetodontidae | *Chaetodon adiergastos* | Corallivore |
|  | *Chaetodon auriga* | Corallivore |
|  | *Chaetodon baronessa* | Corallivore |
|  | *Chaetodon bennetti* | Corallivore |
|  | *Chaetodon ephippium* | Corallivore |
|  | *Chaetodon kleinii* | Corallivore |
|  | *Chaetodon lunula* | Corallivore |
|  | *Chaetodon melannotus* | Corallivore |
|  | *Chaetodon meyeri* | Corallivore |
|  | *Chaetodon ornatissimus* | Corallivore |
|  | *Chaetodon punctatofasciatus* | Corallivore |
|  | *Chaetodon rafflesii* | Corallivore |
|  | *Chaetodon semeion* | Corallivore |
|  | *Chaetodon speculum* | Corallivore |
|  | *Chaetodon trifascialis* | Corallivore |
|  | *Chaetodon trifasciatus* | Corallivore |
|  | *Chaetodon ulietensis* | Corallivore |
|  | *Chaetodon unimaculatus* | Corallivore |
|  | *Chaetodon vagabundus* | Corallivore |
|  | *Forcipiger flavissimus* | Carnivore |
|  | *Forcipiger longirostris* | Carnivore |
| Epinephelidae | *Plectropomus areolatus* | Carnivore |
|  | *Plectropomus laevis* | Carnivore |
|  | *Plectropomus oligacanthus* | Carnivore |
|  | *Variola louti* | Carnivore |
| Labridae | *Cheilinus fasciatus* | Carnivore |
|  | *Cheilinus undulatus* | Carnivore |
|  | *Coris aygula* | Carnivore |
|  | *Coris gaimard* | Carnivore |
|  | *Epibulus insidiator* | Carnivore |
|  | *Gomphosus varius* | Carnivore |
|  | *Halichoeres hortulanus* | Carnivore |
|  | *Hemigymnus fasciatus* | Carnivore |
|  | *Hemigymnus melapterus* | Carnivore |
|  | *Oxycheilinus digrammus* | Carnivore |
|  | *Oxycheilinus unifasciatus* | Carnivore |
| Lethrinidae | *Gnathodentex aureolineatus* | Carnivore |
|  | *Lethrinus erythracanthus* | Carnivore |
|  | *Monotaxis grandoculis* | Carnivore |
| Lutjanidae | *Lutjanus bohar* | Carnivore |
|  | *Lutjanus decussatus* | Carnivore |
|  | *Lutjanus fulvus* | Carnivore |
|  | *Lutjanus gibbus* | Carnivore |
|  | *Lutjanus kasmira* | Carnivore |
|  | *Macolor spp* | Planktivore |
| Pomacentridae | *Amblyglyphidodon aureus* | Planktivore |
|  | *Amblyglyphidodon curacao* | Herbivore |
|  | *Amblyglyphidodon leucogaster* | Herbivore |
|  | *Amphiprion clarkii* | Herbivore |
|  | *Chromis amboinensis* | Planktivore |
|  | *Chromis atripectoralis* | Planktivore |
|  | *Chromis atripes* | Planktivore |
|  | *Chromis lepidolepis* | Planktivore |
|  | *Chromis lineata* | Planktivore |
|  | *Chromis margaritifer* | Planktivore |
|  | *Chromis ternatensis* | Planktivore |
|  | *Chromis weberi* | Planktivore |
|  | *Chromis xanthura* | Planktivore |
|  | *Chrysiptera rex* | Herbivore |
|  | *Dascyllus trimaculatus* | Planktivore |
|  | *Plectroglyphidodon dickii* | Herbivore |
|  | *Plectroglyphidodon johnstonianus* | Corallivore |
|  | *Plectroglyphidodon lacrymatus* | Herbivore |
|  | *Pomacentrus bankanensis* | Herbivore |
|  | *Pomacentrus coelestis* | Herbivore |
|  | *Pomacentrus lepidogenys* | Herbivore |
|  | *Pomacentrus moluccensis* | Herbivore |
|  | *Pomacentrus philippinus* | Planktivore |
|  | *Pomacentrus vaiuli* | Herbivore |
|  | *Pomachromis richardsoni* | Planktivore |
| Scarinae | *Cetoscarus bicolor* | Herbivore |
|  | *Chlorurus bleekeri* | Herbivore |
|  | *Chlorurus microrhinos* | Herbivore |
|  | *Chlorurus sordidus* | Herbivore |
|  | *Hipposcarus longiceps* | Herbivore |
|  | *Scarus dimidiatus* | Herbivore |
|  | *Scarus forsteni* | Herbivore |
|  | *Scarus frenatus* | Herbivore |
|  | *Scarus globiceps* | Herbivore |
|  | *Scarus niger* | Herbivore |
|  | *Scarus oviceps* | Herbivore |
|  | *Scarus prasiognathos* | Herbivore |
|  | *Scarus psittacus* | Herbivore |
|  | *Scarus rubroviolaceus* | Herbivore |
|  | *Scarus schlegeli* | Herbivore |
|  | *Scarus spinus* | Herbivore |
| Siganidae | *Siganus corallinus* | Herbivore |
|  | *Siganus puellus* | Herbivore |
|  | *Siganus punctatissimus* | Herbivore |
|  | *Siganus punctatus* | Herbivore |
|  | *Siganus vulpinus* | Herbivore |
| Zanclidae | *Zanclus cornutus* | Carnivore |
