# Supplementary material for: Caught in the Middle: Combined Impacts of Shark Removal and Coral Loss on the Fish Communities of Coral Reefs
Source: PLoS One. 2013 Sep 18;8(9):e74648. doi: 10.1371/journal.pone.0074648 (PMC3776739; doi:10.1371/journal.pone.0074648)
Supplement: Table S3 — Summary of statistical tests to evaluate fishing, disturbance and interactive effects on densities of trophic groups. Fishing, disturbance and interaction effects were evaluated using a permuted two-way ANOVA. Permuted t-tests were used to conduct contrasts. p-values were Bonferroni corrected. (DOCX) [file pone.0074648.s006.docx]

**Table S3.**

| Trophic Group |  | Test-statistic |  | *p*-value |  | Contrast (permuted *t-*test) |
| --- | --- | --- | --- | --- | --- | --- |
| **Fishing Effects** | | | | | | |
| Carnivore |  | *F*_1,122_ = 13.95 |  | 0.0125 |  | Disturbed=Non-Disturbed |
| Corallivore |  | *F*_1,122_ = 0.0015 |  | 1 |  |  |
| Planktivore |  | *F*_1,122_ = 0.026 |  | 1 |  |  |
| Herbivore |  | *F*_1,122_ = 0.74 |  | 1 |  |  |
| Detritivore |  | *F*_1,122_ = 10.41 |  | 0.001 |  |  |
| **Disturbance Effects** | | | | | | |
| Carnivore |  | *F*_1,122_ = 2.26 |  | 1 |  |  |
| Corallivore |  | *F*_1,122_ = 13.66 |  | 0.0075 |  | Fished=Non-Fished |
| Planktivore |  | *F*_1,122_ = 70.25 |  | 0.0025 |  | Fished=Non-Fished |
| Herbivore |  | *F*_1,122_ = 80.05 |  | 0.0025 |  | Fished<Non-Fished* |
| Detritivore |  | *F*_1,122_ = 35.75 |  | 0.0001 |  |  |
| **Interaction Effects (Fishing x Disturbance)** | | | | | | |
| Carnivore |  | *F*_1,122_ = 0.11 |  | 1 |  |  |
| Corallivore |  | *F*_1,122_ = 2.81 |  | 1 |  |  |
| Planktivore |  | *F*_1,122_ = 0.40 |  | 1 |  |  |
| Herbivore |  | *F*_1,122_ = 2.15 |  | 1 |  |  |
| Detritivore |  | *F*_1,122_ = 15.88 |  | 0.005 |  |  |

*Contrast significant only during disturbed phase (*t*_47_ = 3.62, *p* = 0.001)
