# Supplementary material for: Caught in the Middle: Combined Impacts of Shark Removal and Coral Loss on the Fish Communities of Coral Reefs
Source: PLoS One. 2013 Sep 18;8(9):e74648. doi: 10.1371/journal.pone.0074648 (PMC3776739; doi:10.1371/journal.pone.0074648)
Supplement: Text S1 — (DOCX) [file pone.0074648.s007.docx]

**Artisanal Fishing in the MoU74 Box**

The Australian-Indonesian Memorandum of Understanding Box 74 (MoU74) is an area where Indonesian fishermen are granted access to the Australian Exclusive Economic Zone to pursue fishing using traditional artisanal techniques [1]. Indonesian fishermen target “banquet” species of high economic value, principally shark (for shark fin), but also sea cucumber (*Holothuroidea* *spp.***)** and trochus shell (top snails, *Trochidae* *spp.*) [1-3]. Studies conducted in 1998 corroborate our findings that fisheries activities in the MoU74 have reduced abundances of reef sharks (*Carcharhinidae*) [3]. Recent studies in 2010 and 2011 confirm that this pattern has persisted to the present day [4,5].

Indonesian fishing techniques include snorkelling in shallow water for sea cucumber and top snails and longlining for sharks. Longlines are set at least 50 m off the reef edge to target sharks and avoid entanglement with corals [3]. Due to the distances to markets and a lack of on-board refrigeration, products are generally dried for transportation. Fishing for finfish other than sharks is generally limited to species that are caught for immediate consumption. Significant depletion of finfish stocks (other than sharks) by these fishermen was not detectable in ours or previous studies [3]. Australian Customs and border patrol flights (2000-2007) confirm the presence of Indonesian fishermen in the vicinity of the MoU74 Box throughout the period of the study, with a peak number of vessels spotted in the year 2006 [1].

**Physical and biological differences between Scott Reefs and Rowley Shoals**

Physical, biological and anthropogenic factors that may have contributed to the differences we observed in fish communities between the Scott Reefs and Rowley Shoals have been summarized in Table S1. Fishing is the main difference in anthropogenic activities between these reefs. All reefs are uninhabited and both have a similar area (Scott = 180 km^2^; Rowley = 174 km^2^), although they do differ in perimeters (Scott = 184 km; Rowley = 118 km; Table S1). However, a large portion of the perimeter of South Scott Reef is effectively lagoon due to the horseshoe shape of the reef. Long-term monitoring sites within this lagoon were not included in our study. Small differences in chlorophyll-a concentration and sea surface temperature (reefs at the Rowley Shoals are on average one degree cooler than the Scott Reefs) may exist (Table S1), however, given the resolution of chlorophyll-a measurements (pixel dimensions of 4km x 4km in remote sensing data), such estimates must be treated with caution. Furthermore, there is little evidence of differences in reef productivity since both reefs supported similar mean abundances of fish, coral cover and algal cover when compared over the entire sampling period (1994 – 2008; Table S1). The most striking difference occurred in species diversity, with the Scott Reefs having a greater number of species than the Rowley Shoals (Table S1). All the species that occurred exclusively at Scott Reefs were very rare, occurring in less than 5% of transects. This difference reflects a latitudinal gradient of increasing species richness towards Indonesia and the Coral Triangle [6], a pattern recorded by other studies [7,8].

**Benthic Disturbances**

Both the Scott Reefs and Rowley Shoals experienced catastrophic pulse disturbances in the late 1990s (coral bleaching in 1998 and a Category 5 cyclone in 1996, respectively). At the Scott Reefs, bleaching reduced coral cover from *c.* 60% to <10%, while similar declines in coral cover occurred at two of three reefs of the Rowley Shoals after a Category 5 cyclone (Figure 2A and 2B). After the disturbance, corals were overgrown by turfing algae, but coral cover returned to near pre-disturbance levels for both reefs in the following decade.

Coral bleaching and cyclones are both acute, pulse disturbances that ultimately convert live coral to algal cover. There are, however, also some differences between these events. For example, cyclones physically break and reduce coral structure while bleaching leaves coral skeletons intact, at least for some months after the initial disturbance [9-11]. Ultimately, bleaching also leads to the loss of coral structure as the dead coral is subject to bioeroding organisms and is reduced to rubble, a process that typically requires some months depending on the exposure of a reef to wave action. Despite each having a different initial effect on the structural complexity (or three-dimensional structure) of reefs, studies that have monitored these disturbances have found that both ultimately have very similar effects on fish communities. For example, Wilson *et al.* (2006) found that of five trophic groups of reef fishes (carnivore, herbivore, detritivore, planktivore and corallivore), only planktivores differed in abundance between bleaching and cyclone events. Further, rates of recovery of coral cover after bleaching and cyclonic events also appear to be unrelated to disturbance type [12,13].

In order to determine if there were major differences in the composition of benthic communities between reef systems that might have contributed to our results, we compared communities among treatments (fished, non-fished, disturbed, undisturbed) using Principal Components Analysis (PCA). We summarized differences in benthic communities using 19 different categories of benthic cover. Cover types were chosen based on their abundance and whether they were thought to be important to the habitat and dietary requirements of reef fishes. These categories were: soft coral, branching *Acropora*, digitate *Acropora*, corymbose *Acropora*, bottlebrush *Acropora*, tabulate *Acropora*, *Isopora* brooders, *Montipora*, other encrusting coral, massive coral, Pocilloporidae, foliose, macroalgae, turf/coralline algae, algae other, sand/rubble, sponge, other coral (mostly submassive coral) and other (e.g. invertebrates) [14]. Prior to analysis the percent cover of each was arcsine and Hellenger transformed [15]. This procedure used the *vegan* package of R Statistical Computing [16].

The PCA was able to explain 60.5% of variation in benthic community composition on the first two axes (Figure S1). More than two-thirds of this variation (44.7%) was explained across the first axis and was attributable to differences in algal (turf-crustose) and coral cover (hard and soft), such that sites following a pulse disturbance had more algae and less coral cover than sites prior to disturbance (Figure S1). This analysis also showed that sites at the Scott Reefs suffered greater loss of coral cover and as a result had more algal growth after bleaching than those that underwent the cyclone at the Rowley Shoals (Figure S1). Prior to disturbance both reef systems appeared to have similar amounts of algal and coral cover, although some differences could be seen between fished and non-fished sites.

Differences in types of coral cover between reef systems contributed much less to overall patterns along the second axis of variation (15.8%; Figure S1). There was also a large degree of overlap among the treatments along the second axis of variation (especially for non-disturbed sites). Regardless, there were some differences in coral communities between fished and non-fished reefs under both disturbed and non-disturbed conditions. These were mainly attributable to differences in the cover of macro algae, *Isopora*, sponge and corymbose *Acropora* (Figure S1). While such differences in habitat might be expected to contribute to differences in the fish community between reef systems, there was little evidence for any such effects at least at the level of trophic groups. For example, the Scott Reefs appear to have more macro algae under non-disturbed conditions (Figure S1), a feature that should be expected to benefit abundances of herbivores, when in fact it did not (Figure 4 and S4). Thus, the principal variables in benthic composition that contributed to patterns of fish assemblages at the scale of our study appeared to be related to differences in cover of algae and corals.

**Fish Community Composition Analysis**

Prior to analysis, we removed rare species (defined as present in <5% of sites) [17] and Hellenger transformed abundances [15]. Collinearity between variables was assessed using variance inflation factors (VIF; VIF < 10 indicates non-collinearity). The significance of the Redundancy Analysis (RDA) model, RDA constraints and RDA axes were tested using a randomization procedure (*n* = 9999) where the data were permuted randomly and refitted to test for significance. This procedure involved using the *cca* and *anova* functions in the vegan package of R Statistical Computing [16].

The RDA separated fished and non-fished reefs into two distinct groups (Figure 3). Fished sites tended to have higher abundances of carnivores and a lower abundance of herbivores in comparison to non-fished reefs. The overall model was significant (permutation analyses; *p*<0.01), as were all 5 constraints (p<0.01) and the first 4 axes of variation (or components; *p* <0.05). The RDA was able to explain 29.5% of variation (*n* = 114 species). The first axis explained 13.5% of species variation and was related to the abundance of herbivorous fishes. The second axis of variation (which explained 9.5% of species variation) was related to the abundance of carnivores (Figure 3). Other trophic groups (corallivores, detritivores and planktivores) showed changes in abundance that appeared to be more related to changes in benthic community structure than fishing.

Differences in the abundances of carnivores and herbivores between the Scott Reefs and the Rowley Shoals were the result of patterns occurring across multiple families, genera and species (Figures S2, S3 and Table S2). Plots of the results of principal component analyses (PCA) show that differences in the abundances of carnivores (*n*=15 genera, 26 species) between these fished and non-fished reefs could be attributed to representatives of the genus *Lutjanus* and to a lesser extent on the genera *Forcipiger*, *Gnathodentex, Lethrinus, Plectropomus* and *Zanclus*, all of which tended to be more abundant at the Scott Reefs (Figure S2). In contrast, the small wrasses *Gomphosus and Hemigymnus* tended to be more abundant at the protected Rowley Shoals (Figure S2).

For herbivores, differences in the densities between the reef systems were driven by differences in the abundances of 12 genera (Figure S3). In particular, representatives of the genera *Chlorurus, Naso, Pomacentrus* and *Zebrasoma* were more abundant at the Rowley Shoals than the Scott Reefs (Figure S3). In contrast, the genera *Acanthurus, Chrysiptera* *Plectroglyphidodon* and the rabbitfish *Siganus* characterised herbivores at the Scott Reefs (Figure S3).

**Supporting Information References**

1. Field IC, Meekan MG, Buckworth RC, Bradshaw CJA (2009) Protein mining the world's oceans: Australasia as an example of illegal expansion-and-displacement fishing. Fish and Fisheries 10: 323-328.

2. Rees M, Colquhoun J, Smith L, Heyward A (2003) Surveys of Trochus, Holothuria, Giant Clams and the Coral Communities at Ashmore Reef, Cartier Reef and Mermaid Reef:2003 report. Report produced for Environment Australia. Australian Institute of Marine Science, Townsville.

3. Skewes TD, Dennis DM, Jacobs DR, Gordon SR, Taranto TJ, et al. (1999) Survey and Stock Size Estimates of the Shallow Reef (0–15 m deep) and Shoal Area (15–50 m deep) Marine Resources and Habitat Mapping within the Timor Sea MOU74 Box. , Canberra: CSIRO Division of Marine Research.

4. Heyward A, Jones R, Meeuwig J, Burns K, Radford B, et al. (2011) Monitoring Study S5 Banks & Shoals, Montara 2011 Offshore Banks Assessment Survey, Report for PTTEP Australasia (Ashmore Cartier Pty Ltd). Townsville, Australia. 253 p.

5. Heyward A, Jones R, Travers M, Burns K, Suosaari G, et al. (2011) Monitoring Study S6 Corals Reefs, Montara: 2011 Shallow Reef Surveys at Ashmore, Cartier and Seringapatam Reefs. Final Report for PTTEP Australasia (Ashmore Cartier Pty Ltd.). Townsville, Australia. 163 p.

6. Bellwood DR, Hughes TP, Connolly SR, Tanner J (2005) Environmental and geometric constraints on Indo-Pacific coral reef biodiversity. Ecology Letters 8: 643-651.

7. Sandin SA, Smith JE, DeMartini EE, Dinsdale EA, Donner SD, et al. (2008) Baselines and degradation of coral reefs in the Northern Line Islands. PLoS ONE 3: e1548.

8. Williams ID, Richards BL, Sandin SA, Baum JK, Schroeder RE, et al. (2011) Differences in reef fish assemblages between populated and remote reefs spanning multiple archipelagos across the central and western Pacific. Journal of Marine Biology 2011: Article ID 826234.

9. Letourneur Y, Harmelin-Vivien M, Galzin R (1993) Impact of hurricane Firinga on fish community structure on fringing reefs of Reunion Island, S.W. Indian Ocean. Environmental Biology of Fishes 37: 109-120.

10. Pandolfi JM, Connolly SR, Marshall DJ, Cohen AL (2011) Projecting coral reef futures under global warming and ocean acidification. Science 333: 418-422.

11. Wilson SK, Graham NAJ, Pratchett MS, Jones GP, Polunin NVC (2006) Multiple disturbances and the global degradation of coral reefs: are reef fishes at risk or resilient? Global Change Biology 12: 2220-2234.

12. Graham NAJ, Nash KL, Kool JT (2011) Coral reef recovery dynamics in a changing world. Coral Reefs 30: 283-294.

13. Osborne K, Dolman AM, Burgess SC, Johns KA (2011) Disturbance and the Dynamics of Coral Cover on the Great Barrier Reef (1995-2009). Plos One 6.

14. Gilmour JG, Travers MJ, Underwood JN, Markey KL, Ninio R, et al. (2011) Long-term monitoring of shallow water coral and fish communities at Scott Reef. Townsville, Australia: Australian Institute of Marine Science. 157 p.

15. Legendre P, Gallagher ED (2001) Ecologically meaningful transformations for ordination of species data. Oecologia 129: 271-280.

16. Team RDC (2012) R: A Language and Environment for Statistical Computing. 2.15.0 ed. Vienna, Austria: R Foundation for Statistical Computing.

17. Jackson DA, Harvey HH (1989) Biogeographic associations in fish assemblages: Local versus regional processes. Ecology 72: 1314-1324.

18. Hughes TP, Baird AH, Bellwood DR, Card M, Connolly SR, et al. (2003) Climate Change, Human Impacts, and the Resilience of Coral Reefs. Science 301: 929-933.

19. McCulloch M, Fallon S, Wyndham T, Hendy E, Lough J, et al. (2003) Coral record of increased sediment flux to the inner Great Barrier Reef since European settlement. Nature 421: 727-730.

20. Gardner TA, Côté IM, Gill JA, Grant A, Watkinson AR (2003) Long-Term Region-Wide Declines in Caribbean Corals. Science 301: 958-960.

21. Froese R, Pauly D (2011) Fishbase. www.fishbase.org: World Wide Web electronic publication.

**Supporting Information Legends**

**Table S1**. A summary of anthropogenic, reef metrics, environmental and biotic factors at protected and fished reefs. Protected sites included Mermaid, Clerke and Imperieuse Reefs. Fished sites included South and North Scott, Seringapatam and Ashmore Reefs.

**Table S2**. Species composition of the five trophic groups (carnivore, herbivore, detritivore, planktivore and corallivore) used in our study. The list is alphabetical by family and species. Those classified as corallivores included both obligate and facultative coral feeders [11,18]. Herbivores were classified according to Green and Bellwood (2009) while detritivores (including epilithic algal matrix feeders) followed Wilson et al. (2003). Planktivores and carnivores followed Froese & Pauly (2011) [19-21]. Only those species present in more than 5% of sites were included in this list.

**Table S3.** Summary of statistical tests to evaluate fishing, disturbance and interactive effects on densities of trophic groups. Fishing, disturbance and interaction effects were evaluated using a permuted two-way ANOVA. Permuted *t*-tests were used to conduct contrasts. *p*-values were Bonferroni corrected.

**Figure S1.** Principal components analysis of the benthic composition of 19 different classes of coral, algae, sponge, and other benthos among sites. Benthic cover types contributing the most to patterns are denoted in black, while others are shown in the middle of the plot in grey. Sites have been coded by the four treatments (see key). The amount of variation explained by each axis is shown.

**Figure S2.** PCA biplot of fish abundances by genus in the carnivore trophic group. The sites were coded by each of the four treatments and the 15 genera that made up the carnivore group are shown. The amount of variation explained by each axis is shown.

**Figure S3.** PCA biplot of abundances of fish by genus in the herbivore trophic group. The 12 genera that make up the herbivore group are shown on the figure and the sites were coded by each of the four treatments. The amount of variation explained by each axis is shown.
